# Flupyradifurone impairs the processing of sound signals by the ascending interneurons AN1 and AN2 that convey auditory information to the cricket brain

**DOI:** 10.1007/s00359-026-01814-4

**Published:** 2026-05-11

**Authors:** Marcelo Christian, Manuela Nowotny, Stefan Schöneich

**Affiliations:** Institute for Zoology and Evolutionary Research, Friedrich Schiller University Jena, Erbertstraße 1, 07743 Jena, Germany

**Keywords:** Auditory processing, Insecticide, Flupyradifurone, Identified neurons, Insect hearing, Field crickets

## Abstract

Flupyradifurone is a butenolide insecticide with growing agricultural significance as a substitute for classical neonicotinoids, which have been partially banned in the EU and other countries. It also acts as an agonist on acetylcholine receptors in the insect central nervous system, but its neurophysiological effects on individual interneurons are poorly understood. Here, we investigated the effects of sublethal flupyradifurone intoxication on the neural processing of auditory information in the field cricket *Gryllus bimaculatus*. Using a suction electrode on the brain surface, we recorded the spiking activity of the two ascending auditory interneurons AN1 and AN2 while injecting the insecticide into the prothorax. Flupyradifurone caused a dose-dependent decrease in sound-evoked spike responses and an increase in spontaneous spiking activity in both ascending auditory interneurons, eventually leading up to a collapse of all spiking activity the highest dose tested (20 µl of 10^− 3^ mol/l). The substantial increase of spontaneous spiking activity in AN1 and AN2, which started with 20 µl of 10^− 5^ mol/l flupyradifurone treatment, will mask the neuronal coding of relevant acoustic signals required for auditory feature detection in the higher brain circuits. Field crickets rely on AN1 to transmit low-frequency auditory information for pattern recognition in phonotactic mate finding, and on AN2 to transmit high-frequency auditory information for sound source localization in predator avoidance behaviour. The disruption of auditory processing at the level of ascending interneurons by this insecticide will negatively impact the chances for reproduction and survival in crickets.

**Supplementary Information:**

The online version contains supplementary material available at 10.1007/s00359-026-01814-4.

## Introduction

Pesticides are an integral part of modern agriculture (Jeschke 2016). With extensive monoculture areas as the predominant way of growing crops, there is a steady increase in the worldwide usage of pesticides and especially insecticides (FAO 2024). Modern insecticides, such as the globally most prevalent neonicotinoids, had been designed to specifically target insects and are therefore less harmful for other animals, especially vertebrates including human (Matsuda et al. 2001). Nevertheless, they can also negatively impact a wide range of non-target insects, including beneficial pollinators, as they do not act selectively on pest species only (Desneux et al. 2007; Müller 2018). The soaring use of neonicotinoid insecticides is considered one of the reasons for the dramatic decline in insect fauna over the last decades (Godfray et al. 2014; Hallmann et al. 2017; Cardoso et al. 2020). A potentially more bee-friendly substitute for classical neonicotinoids, which are now partly banned in some countries (Dentzman et al. 2025), is the new butenolide insecticide flupyradifurone (Nauen et al. 2015; Gao et al. 2026). Flupyradifurone is currently gaining significant agricultural relevance as it may also overcome the partial efficacy loss of the most frequently used insecticides due to substance-specific resistances developed by some pest insects (Bass et al. 2015; Matsuda et al. 2020). These recent trends are reflected by a rising number of new research studies investigating potential side effects of flupyradifurone for non-target insects (Tan et al. 2017; Bartlett et al. 2018; Hesselbach and Scheiner 2018, 2019; Al Naggar and Baer 2019; Hesselbach et al. 2020; Tosi et al. 2021; Scheibli et al. 2023, 2024; Lima et al. 2024; Schöfer et al. 2025; Kline et al. 2025; Góngora-Gamboa et al. 2025; You et al. 2026; Dong et al. 2026).

Similar to classical neonicotinoids, flupyradifurone also acts as a competitive agonist with a strong binding affinity to nicotinic acetylcholine-receptors (nAChRs) in the central nervous system of insects (Tan et al. 2007; Casida 2018). It primarily causes depolarisation in the dendrites of interneurons with cholinergic input synapses and thereby furthers the generation of action potentials (Buckingham et al. 1997). Severe intoxication leads to muscle spasms, whole body paralysis and eventually the death of the insect (Suchail et al. 2001; Scheibli et al. 2023; Christian et al. 2025). For sublethal dosages it was shown that flupyradifurone can negatively affect learning, memory and cognition, motor behaviours, brood care, reproduction and foraging in bees and bumble bees (Hesselbach and Scheiner 2018, 2019; Hesselbach et al. 2020; Siviter and Muth 2022; Fischer et al. 2023; Richardson et al. 2024; You et al. 2026). The initial research efforts mainly focused on hymenopteran insects, arguably being the economically most important pollinators (Khalifa et al. 2021). Beside bees, there is now also an accumulating number of studies that report negative effects on other non-target insects, e.g. on the mobility and locomotion in mayfly larvae, lacewings and lady beetles, indirect feeding effects in predatory bugs, and host finding in parasitoid wasps (Bartlett et al. 2018; Scheibli et al. 2023, 2024; Lima et al. 2024; Schöfer et al. 2025). Such complex insect behaviours require sensory perception and further neuronal processing of visual, olfactory, tactile and/or auditory cues. In locusts it was shown that cholinergic insecticides affect the visual processing that is crucial for collision avoidance behaviour (Parkinson et al. 2017) and impair the auditory processing (Christian et al. 2025). They also influence chemical communication in moths by altering the pheromone sensitivity in olfactory interneurons and reducing the pheromone production (Rabhi et al. 2016; Navarro-Roldán and Gemeno 2017). In parasitoid wasps, cholinergic insecticides impair the sex pheromone perception, mate finding, courtship and copulation behaviours (Wang et al. 2018; Schöfer et al. 2025). Besides these chemical communication systems (Müller 2018), hitherto little is known about the potential impact of cholinergic insecticides like flupyradifurone on the neural processing in other sensory modalities used for mate finding. Moreover, since previous studies focused mostly on lethality and the observation of sub-lethal behavioural impairments, there is still a lack of knowledge about the neurophysiological effects of these insecticides at the level of individual interneurons of the neural circuits underlying the various behaviours.

Field crickets are a well-studied neuroethological model organism for auditory processing underlying predator avoidance and mate finding (Marsat and Pollack 2010; Schöneich 2020). The males produce a species-specific calling song with a relatively low sound frequency (~ 5 kHz) to attract conspecific females, which then phonotactically approach the singing male (Hedwig 2006). In contrast, high-frequency sounds like the echolocation signals of insectivorous bats elicit phonotactic avoidance reactions (Wyttenbach et al. 1996; Marsat and Pollack 2012). In field crickets, the ability to perceive sound signals is based on about 50 sensory neurons of the tympanic ears positioned in the tibia of the front legs. The majority (~ 75%) of these auditory receptors are narrowly tuned to the low frequency of the species-specific call, while the remaining are more broadly tuned to higher (10–40 kHz) sound frequencies (Imaizumi and Pollack 1999). Their afferent axons project through the auditory nerve into the prothoracic ganglion where they ramify and synapse with a small number of identified first-order auditory interneurons (Pollack and Hedwig 2017). In each body side, field crickets have two bilateral mirror-image pairs of local omega neurons (ON1 and ON2) and two ascending auditory interneurons (AN1 and AN2) in the prothoracic ganglion (Wohlers and Huber 1982). The ON1 neuron gets excitatory input from low- and high-frequency afferents of the ipsilateral ear (Faulkes and Pollack 2001) and inhibits its contralateral mirror neuron as well as the two ANs (Selverston et al. 1985; Horseman and Huber 1994; Hirtz and Wiese 1997; Faulkes and Pollack 2000), which receive monosynaptic excitation from the afferents of the other ear (Hennig 1988). AN1 is sharply tuned to the low-frequency sound of the cricket calling song (~ 5 kHz) and is essential for phonotactic mate finding based on species-specific pulse pattern recognition by higher processing in a small network of local brain neurons (Schöneich et al. 2015). AN2, on the other hand, is most sensitive to high-frequency sound (> 10 kHz) and forwards its spike responses to the brain for the detection of potentially dangerous sounds signals like the ultrasonic calls of echolocating bats (Fullard et al. 2005; Marsat and Pollack 2012).

Both, AN1 and AN2, have ascending axons from the prothoracic ganglion to the brain, where their axonal terminals ramify in a ring-like auditory neuropile (Schöneich et al. 2015). As the morphological structure and physiological response characteristics of these individually identified auditory interneurons are well studied (Wohlers and Huber 1982; Schöneich 2020), crickets present an excellent model organism to investigate the effect of flupyradifurone on their auditory pathway at the cellular level. Here, we used suction electrodes at the brain surface of field crickets to extracellularly measure the spiking activity of AN1 and AN2 (Kostarakos and Hedwig 2017). Due to the superficial position of their axonal branches in the ventral protocerebrum, this method enables long lasting recordings of these two interneurons and allows the comparison of increasing concentrations of flupyradifurone in the same recording over time.

In a previous study with locusts (Christian et al. 2025) we have shown that flupyradifurone does not affect the responses of the sensory afferents in the auditory nerve but drastically reduced the spike responses across the population of 20–25 auditory interneurons with ascending axons in the neck connectives. Studying now crickets, which have evolved their ears and auditory pathway independently of locusts, will give insight on how general the previously described effects are for different insect groups. Moreover, in field crickets it is possible to selectively record the spike activity of AN1 and AN2, two individually identified ascending interneurons with demonstrated relevance for different auditory behaviours (Schöneich 2020).

## Materials and methods

### Animals

In this study we used adult Mediterranean field crickets (*Gryllus bimaculatus* deGeer) of both sexes from our laboratory breeding stock at the Zoological Institute in Jena. The fresh body mass of the animals was 1.4 ± 0.5 g (mean ± min/max). Animals were housed in plastic terraria under crowded conditions with constant temperature of 25 ± 2 °C, 40–50% humidity, a 12:12 h light: dark cycle and were fed with fish food, oat flakes and fresh carrots *ad libitum*. The experiments were carried out at room temperature (20–25 °C) and complied with the principles of laboratory animal care and the animal protection laws and regulations of Germany and the European Union.

### Electrophysiology

In preparation for the recordings, the hindlegs and antennae were removed, and the cricket was fixed dorsal side up on a plasticine-covered animal holder by restraining the body and middle legs with fitting metal clamps. The front legs with the tympanic ears were carefully fixed with metal clamps. In each animal we visually checked that the acoustic spiracle was still opened in the same way as in a natural standing posture. The head was fixed with insect needles, and a small window was cut in the head capsule centrally between the eyes. Trachea and fat tissue were carefully removed to expose the brain. The cuticle of the pronotum was punctured on one side to make a hole for ringer and drug injections into the haemolymph surrounding the prothoracic ganglion. To prevent the nervous tissue from drying out, the exposed brain was rinsed with insect ringer (NaCl 0.7%; KCl 0.02%; CaCl_2_ 0.02%; NaHCO_3_ 0.004%; pH 7.4).

**Fig. 1:**
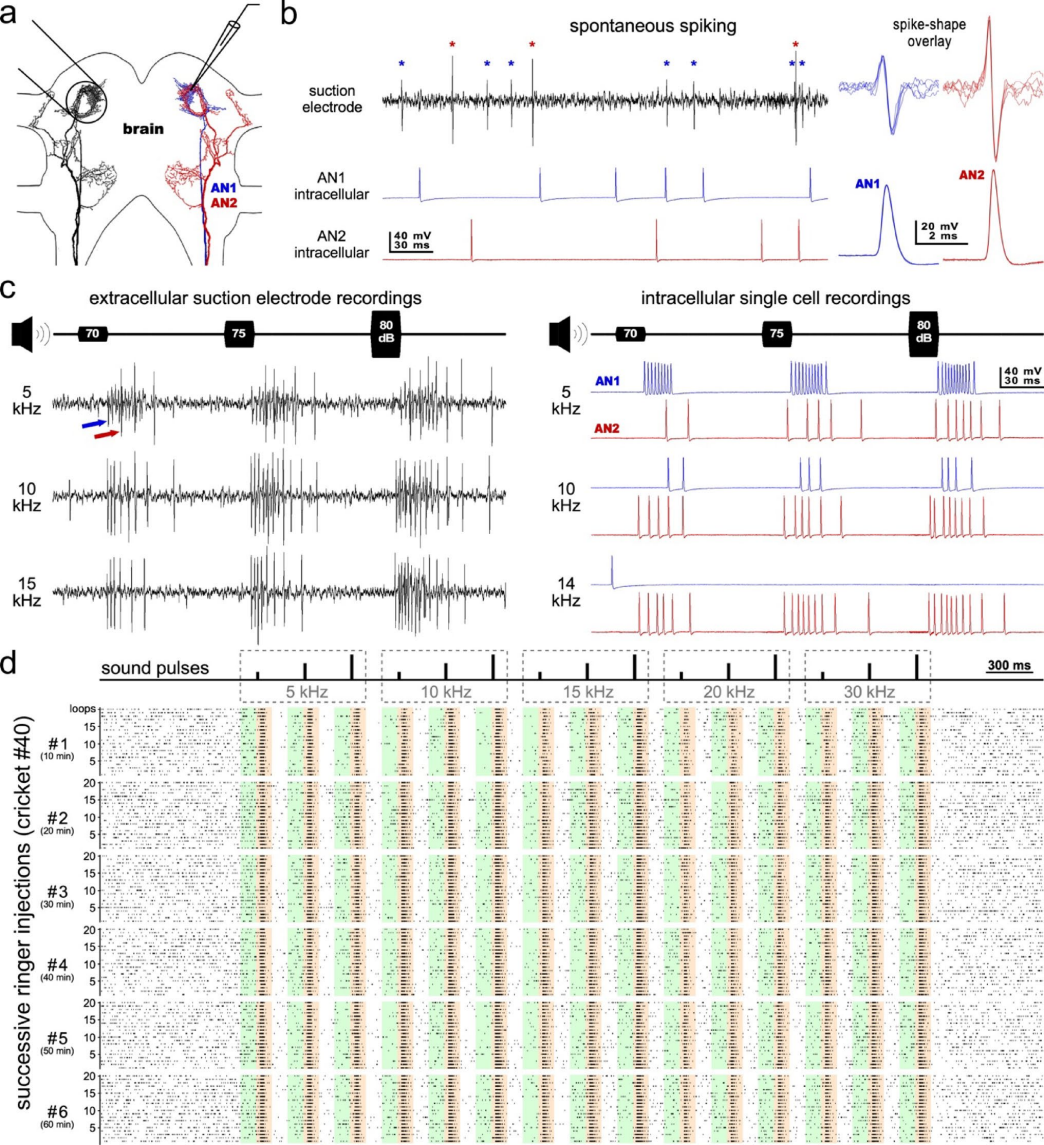
Brain surface suction electrode recordings of spike activity in the ascending auditory interneurons AN1 and AN2. **a** Pictogram of the cricket brain with the electrode positions for independent extracellular (left) and intracellular (right) recordings in relation to the axonal arborizations of AN1 (blue) and AN2 (red) in the ring-like auditory neuropile of the protocerebrum. Drawing of neuronal arborization adapted from ter Hofstede et al. (2015). **b** Spontaneous spiking activity measured without sound stimulation. Left: Extracellular suction electrode recordings (top) and intracellular recordings of AN1 and AN2 (bottom). Right: Overlay of 5 spikes each. **c** Extracellular suction electrode (left) and intracellular single cell (right) recordings of AN1 and AN2 spikes in response to different sound frequencies and amplitudes. In our suction electrode recordings, there are two distinguishable large-amplitude spike units that relate to AN1 (blue arrow) and AN2 (red arrow) based on their spike response properties revealed by intracellular single cell recordings. **d** Exemplary raster plot of suction electrode recording in one control animal with 6 consecutive ringer injections at 10 min intervals. Shown are 20 stimulation loops per treatment, each containing a total of 15 sound pulses (70, 75 and 80 dB SPL for each tested frequency) and each dash represents a single spike of either AN1 or AN2. Highlighted are the 100 ms before (green) and after (orange) the stimulus onset. Note that the spike responses to the sound stimuli remained consistent over the time of the experiment

We used a suction electrode at the brain surface to extracellularly record the spiking activity of AN1 and AN2 (Kostarakos and Hedwig 2017). The self-made device was composed of an electrode holder (type: MEH900R, WPI, Sarasota, FL, USA) with a piece of plastic tubing (1.0/0.5 mm outer/inner diameter, Portex, UK) that was heated to soften and then hand pulled and cut with a scalpel to produce a fine tip (0.2 mm diameter at the end) with smooth end. A piece of silver wire (0.2 mm diameter) inside the ringer-filled electrode tip secured the electrical conduction and a second silver wire in the haemolymph next to the electrode tip served as the reference electrode. Using a micromanipulator (UM-3 C, Narishige, Japan) the electrode was carefully positioned on the ventral side of the protocerebrum (Fig. 1a), and the brain surface was sucked onto the tip by gently applying low pressure with a connected syringe. With a good seal the recording was then stable for several hours. The electrical signal was band-pass filtered (10–10000 Hz) and amplified (1000×) with a differential amplifier (Model 1700, A-M Systems, Carlsborg, WA, USA) before it was digitized and stored with 20 kHz sampling rate (A/D-converter Micro1401-4 and Spike2 v.10 software; CED, Cambridge, UK).

To physiologically identify the individual interneurons in our extracellular recordings with the suction electrode, intracellular recordings from the axonal branches of AN1 and AN2 were made with sharp microelectrodes in the brain of one additional cricket (Fig. 1a–c). The detailed methods for the intracellular recordings had been previously described (Schöneich et al. 2015).

### Acoustic stimulation

We generated 15 different pure-tone pulses of 20 ms (with 1 ms rise and fall ramp) at 70, 75 and 80 dB SPL with sound frequencies of 5, 10, 15, 20 and 30 kHz using Audacity (v.3.3, https://audacityteam.org). The stimulation file was organized so that 3 sound pulses of increasing intensity were presented for each of the 5 sound frequencies in ascending order. This stimulation file was then played in loop-mode so that there were always 300 ms from start of a sound pulse to the start of the next one (see Fig. 1d). For the playback we used an external soundcard (fireface 400, RME, Audio AG, Germany) and a broad-band loudspeaker (R2904, ScanSpeak, Vidbæk, Denmark). The speaker was placed at a distance of 20 cm to the cricket, vertically at level with the animal’s body (10 cm elevation over the table) and ipsilateral (at 90° azimuth relative to the body axis) to the recorded brain hemisphere. For each frequency the sound amplitude was calibrated at the position of the animal to 80 ± 2 dB SPL with a free field microphone (MK301, Microtech Gefell GmbH, Germany; sound calibrator type 4231 and measuring amplifier type 2610, Brüel & Kjær, Nærum, Denmark). The experiments were conducted in a sound insulated anechoic chamber.

### Neuropharmacology

We purchased flupyradifurone as salt (Sigma-Aldrich/Merck KGaA, Germany) and dissolved it in distilled water before diluting with insect ringer to create 5 solutions with drug concentration increasing tenfold from 10^− 7^ to 10^− 3^ mol/l (0.1 µmol/l to 1 mmol/l). We used a 50 µl Hamilton syringe (Bonaduz AG, Switzerland) to inject 20 µl of the respective solution into the haemolymph through the pre-punctured hole in the cuticle of the pronotum. Pre-tests with a single injection of 20 µl 10^− 5^ mol/l flupyradifurone revealed that the drug effect was established within 10 min after treatment and did then not significantly change for at least one hour (see Fig. S1). Therefore, in the main experiments we always waited 10 min for the drug to diffuse into the nervous tissue before the recording was started and the sound stimulation was played in loop-mode for at least 2 min (20–25 loops). We repeated this for each drug concentration, starting with insect ringer as the reference measurement, and then subsequently increasing the insecticide dosage in tenfold steps from 10^− 7^ to 10^− 3^ mol/l, 20 µl each. Note that with this treatment regimen, for each subsequent injection there may be an additive drug effect of max. 10% from the previous treatment. We applied the same protocol in a control group with repeated injections of pure insect ringer to compare it with the group of flupyradifurone treated animals.

### Data analysis

The electrophysiological data was analysed using Spike2 (version 10, CED, Cambridge, UK) and RStudio (version 4.1.2, R Core Team 2021). The combined spike activity of AN1 and AN2 were counted by setting a threshold for all large-amplitude spikes in each recording (Fig. 1c). Raster plots that show each counted spiking event were made for 20 stimulation loops per treatment. In total we recorded more than 60 crickets, but included in our analysis only the recordings in which we had clear spike responses to all tested sound frequencies (AN1 response to low and also AN2 response to high frequencies): *N* = 13 (10 females and 3 males) for flupyradifurone group and *N* = 17 (11 females and 6 males) for ringer control group. In 10 of these recordings (5 control and 5 flupyradifurone treated) we were able to identify and count the action potentials of AN1 and AN2 separately, based on the distinctive spike shapes with different amplitudes. In these animals we analysed 5 stimulation loops with the 15 amplitude-frequency combinations for each treatment. The combined (all recordings) or individual (10 recordings) sound-induced spike responses of AN1 and AN2 were calculated respectively by subtracting the spikes within 100 ms before the stimulus (spontaneous spiking window) from the spikes within 100 ms after the start of each sound pulse (response window).

Furthermore, we calculated semi-logarithmic dose-response curves based on a 4-parameter logistic model (‘dr4pl’ R package v2; An et al. 2019) that was fitted with the individual responses of AN1 and AN2 at their respective best frequency to estimate the effective concentration associated with 50% response reduction (EC50) compared to the initial ringer measurement. We further calculated a response-index for AN1 and AN2 at their respective best frequency by dividing the number of spontaneous spikes within the 100 ms before each sound stimulus (s) by the number of spikes within 100 ms starting at the sound pulse onset (r) and subtracted this value from 1 (formula: 1-s/r). The index range is from 1 (only spiking in response to sound stimulation) to -1 (less spiking activity with sound stimulation compared to spontaneous spiking). An index value of 0 indicates equal spiking activity with and without sound stimulation.

Median, interquartile range (IQR) and variance were calculated for data sets that failed testing for normal distribution (Shapiro–Wilk normality test). In pooled data sets, each contributing animal is equally represented (*n* number of data points, *N* number of animals). Measurements of auditory interneuron responses and spontaneous spiking activity were compared pairwise with the corresponding ringer control treatment using the Wilcoxon test (‘rstatix’ R package version 0.7.1, p-values were Benjamini–Hochberg adjusted). All figures were made using the R package ‘ggplot2’ version 3.4.0 (Wickham 2016) and CanvasX-draw (Canvas GFX, Inc., USA).

## Results

In this study we extracellularly measured the spiking activity of the two ascending auditory interneurons AN1 and AN2 in the cricket brain using a surface suction electrode. With this electrophysiological setup we were able to investigate the effects of the cholinergic insecticide flupyradifurone on the auditory processing at the level of two individually identified interneurons in an acoustically communicating insect. In summary, our results show that sub-lethal dosages of flupyradifurone caused a reduction of sound-evoked spike responses and an increase in the spontaneous spiking activity of AN1 and AN2.

**Fig. 2:**
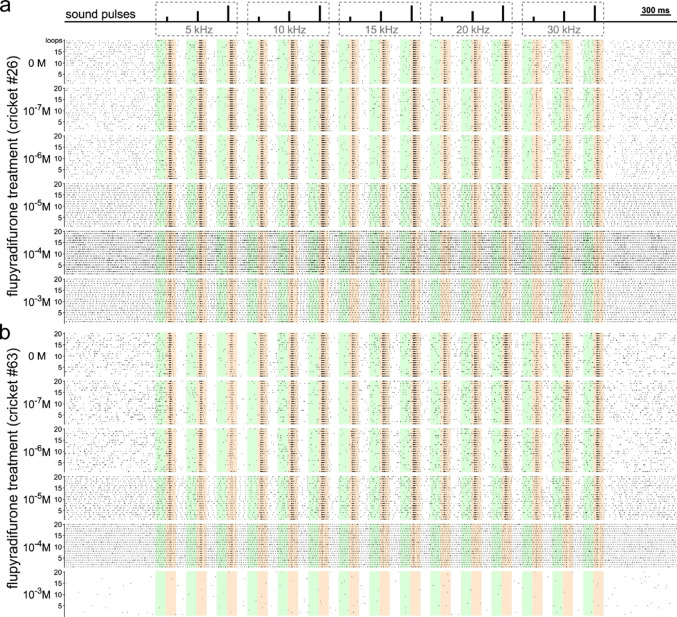
Exemplary raster plots of the AN1/2 spike activity in two crickets of the flupyradifurone treatment group. During the suction electrode recording, each animal received consecutive 20 µl injections of increasing flupyradifurone concentration at 10 min intervals. After injection of 10^− 5^ mol/l flupyradifurone both animals (**a**, **b**) showed a notable increase of the spontaneous spiking activity, that peaked at 10^− 4^ mol/l and decreased again with the highest concentration. The sound-induced spiking responses (highlighted in orange) also decreased at the highest concentrations. Note that for 10^− 3^ mol/l, in one cricket (**a**) there was still spontaneous spiking and also a distinguishable spiking response to the sound stimuli, whereas all spiking activity had almost completely ceased in the other animal (**b**)

Using the same acoustic stimuli, we first compared the typical spike responses in our suction electrode recordings with intracellular recordings of AN1 and AN2 in the brain of one additional cricket (Fig. 1a). Hence, in our suction electrode recordings we could reliably identify the action potentials of the two ascending auditory interneurons AN1 and AN2 as the spikes with distinctively large amplitudes. In some recordings it was possible to clearly differentiate between AN1 and AN2 activity as 2 spike units with different amplitude (Fig. 1b, c). For low-frequency sound (5 kHz), AN1 had its peak spike response in intracellular and suction electrode recordings (Fig. 1c). Both ascending interneurons responded to 10 kHz sound pulses. At higher frequencies (14–15 kHz), almost no stimulus-correlated spike activity was observed for AN1 and AN2 had its peak spike response (Fig. 1c). In contrast to intracellular recordings, the brain surface suction electrode provides the opportunity to reliably record the spiking activity of AN1 and AN2 for hours. Not all of our extracellular recordings allowed to reliably distinguish between the two ascending auditory interneurons by their spike amplitudes. Nevertheless, with a threshold setting covering all large-amplitude spikes in the recording, raster plots show the combined responses and spontaneous activity of both neurons (AN1/2) to sound stimuli of all tested frequency-amplitude combinations with repeated ringer injections (Fig. 1d) and with injections of increasing flupyradifurone dosages (Fig. 2).

**Fig. 3:**
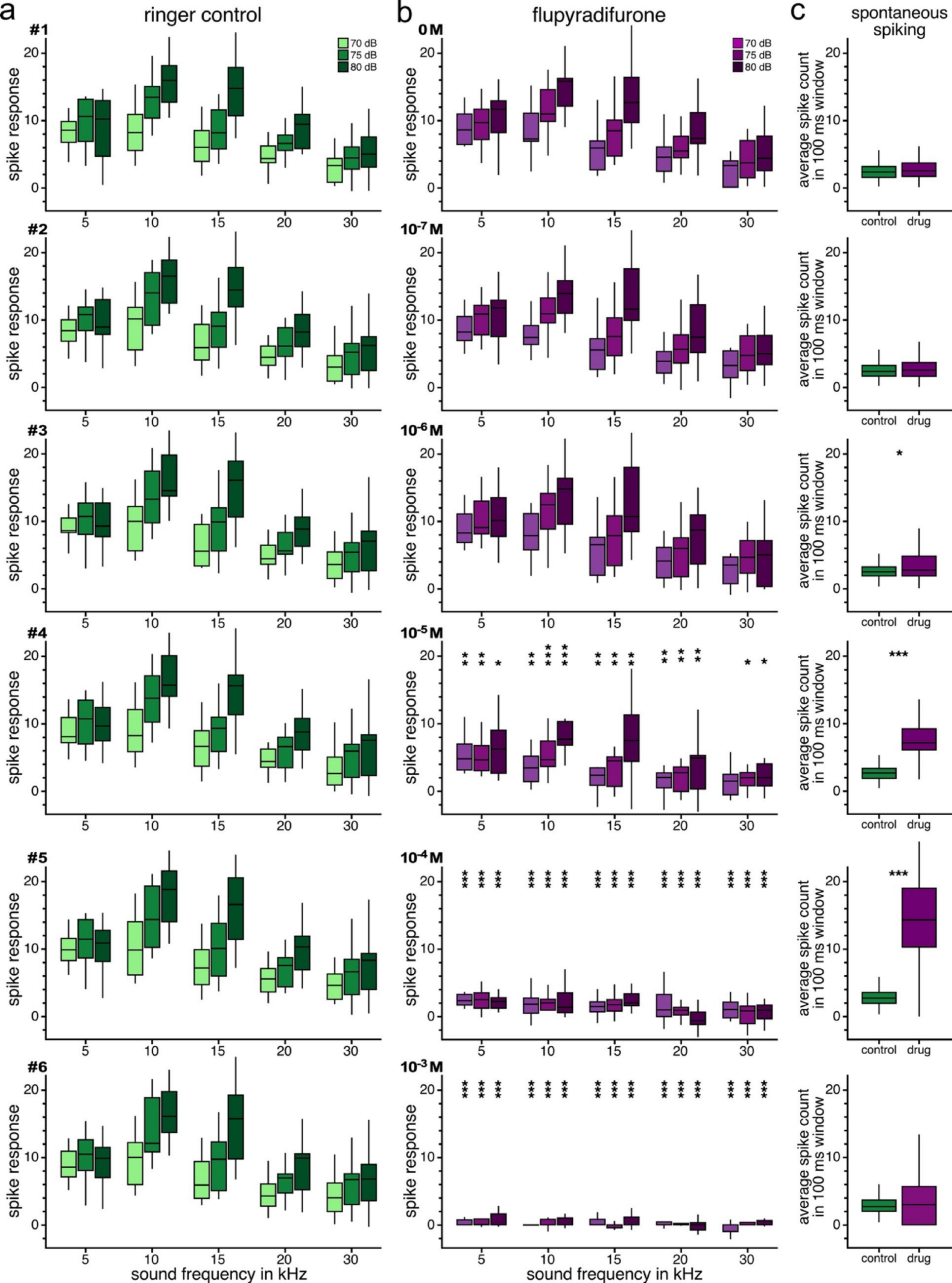
Comparison of AN1/2 spike activity between the ringer control and flupyradifurone treatment group. **a** Spike responses in the ringer control group to 20 ms sound pulses of different sound frequency and amplitude (*N* = 17). For each stimulus (frequency-amplitude combination) the spike responses did not significantly change over the duration of the experiment (see from top to bottom row). **b** Spike responses in the flupyradifurone group (*N* = 13). In comparison with the respective ringer control measurements, the spike responses decreased significantly for all frequencies after injection of 10^− 5^ mol/l of flupyradifurone and higher dosages. **c** Spontaneous spiking activity in the ringer control group (green, *N* = 17) remained consistent over the time of the experiment. In comparison to the ringer control experiments, the spontaneous spiking activity in the flupyradifurone treatment group (purple, *N* = 13) significantly increased with 10^− 6^ mol/l flupyradifurone treatment, peaked at 10^− 4^ mol/l and decreased again for the highest concentration. Boxplots show median (horizontal line), interquartile range (box) and data distribution within 1.5 times of the IQR (whiskers). Asterisks indicate significant differences (*p* < 0.05*, *p* < 0.01**, *p* < 0.001***), always compared to the corresponding ringer control measurement (Wilcoxon tests with Benjamini–Hochberg adjusted p-values; see methods for details)

In the ringer control group (*N* = 17; Fig. 3a), the two ascending auditory interneurons (AN1/2) showed consistent spike responses to each individual sound pulse that did not significantly change over the time of the experiment. For stimuli with the same sound frequency there were increased spike responses for higher sound levels, with the exception of 5 kHz for which AN2 is not much responsive and AN1 has its lowest threshold and therefore mostly saturates above 75 dB SPL. With the same sound level, the 10 kHz stimulation evoked the highest combined spike responses (AN1 and AN2 responding both), followed by 5 and 15 kHz (mainly AN1 and mainly AN2 response, respectively), while for 20 and 30 kHz (only AN2 response but less sensitive) we measured the lowest sound evoked spike responses.

The flupyradifurone treatment group (*N* = 13; Fig. 3b) did not differ significantly from the control group (*N* = 17; Fig. 3a) at the starting conditions where both groups had pure ringer injections (all stimuli: *p* > 0.9) and also after the injection of 10^− 7^ mol/l and 10^− 6^ mol/l (all stimuli: *p* > 0.8 and *p* > 0.6, respectively). After injection of 10^− 5^ mol/l flupyradifurone the spike responses were significantly reduced for all tested sound stimuli (*p* < 0.03), with the exception of 30 kHz at 70 dB (*p* = 0.07). For stimuli with the same sound frequency there were still increased spike responses for higher sound amplitudes, except for 5 kHz. High flupyradifurone concentrations (10^− 4^ and 10^− 3^ mol/l) abolished most sound-induced spike responses (significantly different to ringer control for all stimuli: *p* < 0.001) and consequently also the response differences for stimuli with same sound frequency but different levels. For all individual p-values see supplementary material (Tab. S1).

The average spontaneous spiking activity combined for AN1 and AN2 was measured within the 100 ms time window before the onset of the respective sound pulse (Fig. 3c). Spontaneous spiking activity in the ringer control group did not change significantly (25 ± 2 spikes/s; *p* > 0.05 each) over the entire time of the experiment (~ 60 min). The ringer control and drug treatment group did not significantly differ at the starting conditions and also not for the lowest flupyradifurone concentration of 10^− 7^ mol/l (*p* = 0.07 and *p* = 0.3, respectively). Between flupyradifurone treatment with 10^− 7^ mol/l and 10^− 6^ mol/l, there was a small but statistically significant increase in spontaneous spiking activity (*p* = 0.02). After injection of 10^− 5^ mol/l flupyradifurone, there was a strong significant increase in spontaneous spiking activity compared to ringer control (median: 27 vs. 71 spikes/s; *p* < 0.001), which further intensified after injection of 10^− 4^ mol/l flupyradifurone (median: 27 vs. 143 spikes/s; *p* < 0.001). After injection of 10^− 3^ mol/l flupyradifurone, however, the spontaneous spiking activity diminished in most animals so that there was no significant difference to the ringer control group (*p* = 0.38), but with a higher variability between the animals within the flupyradifurone group (IQR: 20–37 vs. 0–57 spikes/s). With this highest dosage tested, we still recorded a relatively high spontaneous spiking activity in about 25% (*N* = 3 of 13) and almost no spontaneous spiking in about 50% (*N* = 6 of 13) of the animals (cf. Figure 2).

**Fig. 4:**
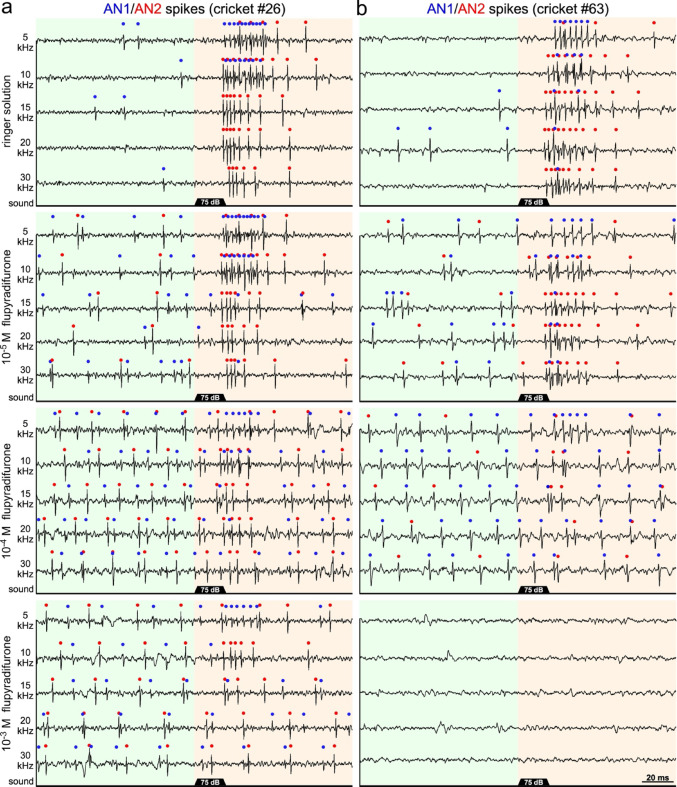
Exemplary recording traces of two flupyradifurone treated crickets in which AN1 and AN2 spikes were clearly distinguishable by a distinct amplitude difference. Shown are only the initial ringer treatment and the three highest insecticide concentrations of the experiment for each tested frequency at 75 dB SPL. The 100 ms before the stimulus are highlighted in green (spontaneous spiking window) and 100 ms after the start of each sound pulse are highlighted in orange (spike response window). Coloured dots mark the individual AN1 (blue) or AN2 (red) spikes. Note that in some recordings the spike amplitude of AN2 is larger than of AN1 (**a**), and in others the AN1 spikes are larger than AN2 spikes (**b**)

**Fig. 5:**
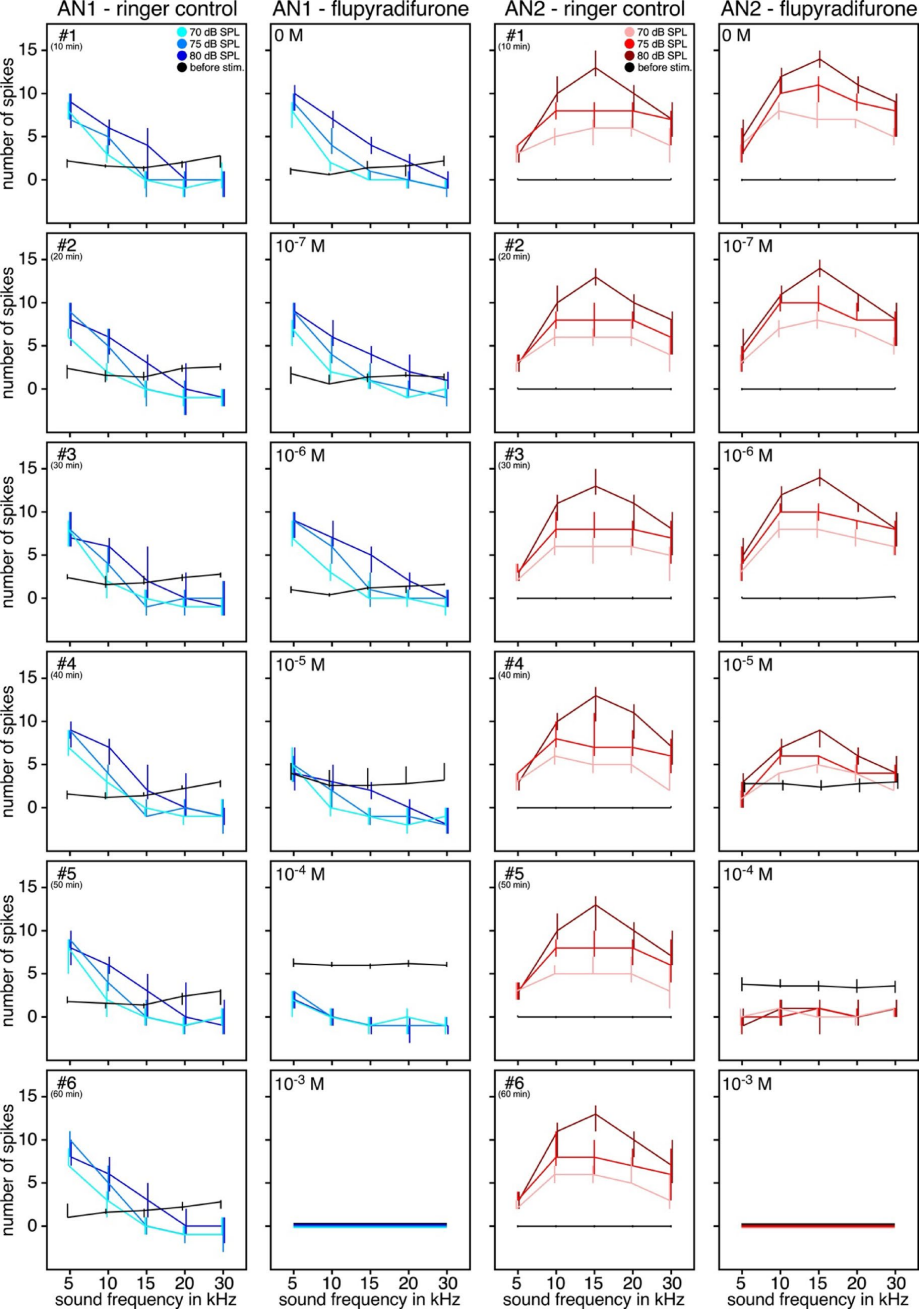
Sound-induced spike responses and spontaneous spike activity of the two individually identified interneurons AN1 and AN2 in flupyradifurone treated and ringer control animals (*N* = 5 each). Shown are the spike responses (median ± IQR) for AN1 (shades of blue) and AN2 (shades of red) to 20 ms sound stimuli of the different amplitude-frequency combinations. Coloured lines connect the data points of same sound pressure level at the different frequencies. Black lines represent the spontaneous spiking activity (median ± IQR) before the sound stimulation. Without flupyradifurone treatment, AN1 had the highest spike response to 5 kHz and AN2 to 15 kHz. With increasing flupyradifurone concentrations (10^− 5^ and 10^− 4^ mol/l), the spontaneous spiking activity of AN1 and AN2 increased while their spike responses to sound stimuli decreased. At the highest concentration tested (10^− 3^ mol/l), there was almost no spontaneous and sound-induced spiking in both neurons, AN1 and AN2. Note that the spontaneous spike activity of AN1 is slightly reduced during the low-frequency stimulation blocks due to activity-dependent habituation effects during the stimulation loop

We analysed the spontaneous activity and sound-induced responses separately for AN1 and AN2 in those recordings where the spikes signals of the two ascending auditory interneurons can be reliably distinguished based on clear differences in shape and amplitude (e.g. Figure 4). This was possible for *N* = 10 of our recorded crickets, *N* = 5 of the ringer control group and *N* = 5 of the flupyradifurone treatment group (Fig. 5). Due to the slight differences in the positioning of the suction electrode at the brain surface of the crickets, the AN2 spikes had the larger amplitude in some recordings (Fig. 4a) whereas in other recordings the AN1 spikes were larger (Fig. 4b). In all recordings, AN1 showed peak spiking activity in response to 5 kHz sound stimuli (medians: 8–9 spikes at 80 dB SPL) and AN2 had the highest spike response to 15 kHz stimuli (medians: 13–14 spikes at 80 dB SPL). Without drug treatment, spontaneous spiking was relatively low and almost exclusively from AN1, while AN2 showed little to no spontaneous activity (Figs. 4, 5). For the highest tested sound frequencies (20 and 30 kHz) within the stimulation loop, there was a small increase of the average spontaneous activity in AN1 (Fig. 5, black line). Overall, for both interneurons the sound pulses with higher sound pressure level evoked stronger spike responses within the range of their respective frequency tuning. Sound-induced spike responses in the ringer control group were consistent over the time of the experiment for both neurons. After injection of 10^− 5^ mol/l of flupyradifurone, the sound-induced spike responses of AN1 and AN2 decreased while the average spontaneous spike activity increased. For 10^− 4^ mol/l, the sound-induced spike responses further decreased in both interneurons. The spontaneous spiking activity of both interneurons further increased, with AN1 having the higher number of spontaneous spikes compared to AN2. When the spontaneous spiking was highest, both interneurons would spike at very consistent intervals (Fig. 4). At the highest tested concentration (10^− 3^ mol/l flupyradifurone), spontaneous spiking and sound-induced responses decreased for both auditory interneurons (Fig. 4a) and in most animals the spiking activity ceased completely (Figs. 4b, 5).

**Fig. 6:**
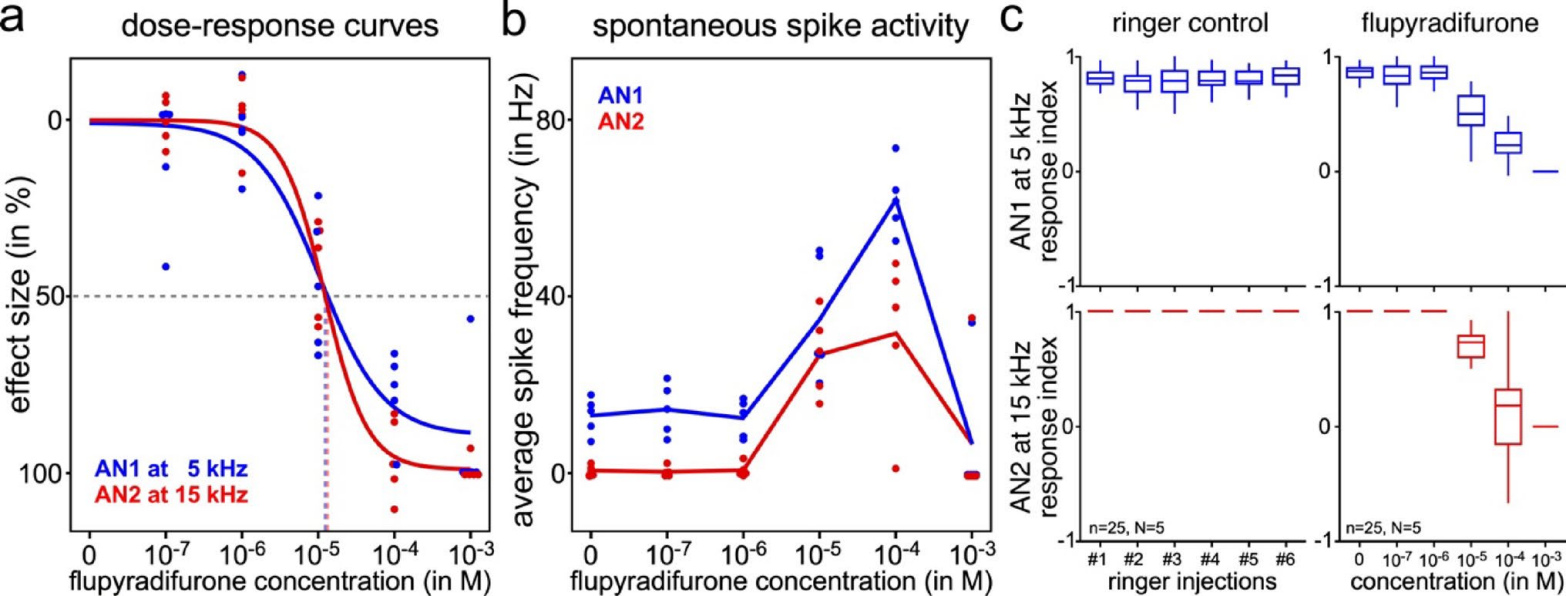
Comparison of the impact of flupyradifurone on the interneurons AN1 and AN2. **a** Dose-response curves showing the effect size of the flupyradifurone treatment on the spike response of AN1 (blue; *N* = 5) and AN2 (red; *N* = 5). The dotted lines indicate the calculated effective concentrations associated with 50% effect magnitude (EC50), which was about 45 ng/g body mass of the crickets for both, AN1 and AN2 (20 µl of about 11 µmol/l) flupyradifurone at their respective best frequency. **b** Dose-dependent changes of the spontaneous spike activity in AN1 (blue) and AN2 (red). Plotted are for both interneurons the individual data points of each animal (*N* = 5) as well as the mean spike activity (coloured lines) for all tested flupyradifurone concentrations. **c** Comparison of the calculated response-indices (auditory responsiveness vs. spontaneous activity) for the two ascending auditory interneurons AN1 and AN2 at their best frequency between ringer control and flupyradifurone treatment group (*N* = 5 each). For both AN1 and AN2, the response-index (see methods) remains relatively constant over the duration of the experiment for ringer control group (left) and decreased for the higher concentrations (> 10^− 6^ mol/l) in the flupyradifurone group (right). Index values close to 1 correspond to strong spike responses to sound and relatively low spontaneous spiking activity during silence; values close to 0 correspond to similar spike numbers of spontaneous activity and sound-induced responses. Boxplots show median (horizontal line), interquartile range (box) and data distribution within 1.5 times of the IQR (whiskers)

To compare the flupyradifurone effects between AN1 and AN2, dose-response curves, spontaneous spiking activity and the calculated response indices for the different treatments are shown (Fig. 6). The data-fitted dose-response curves indicate a very similar reduction of sound-induced spike responses by flupyradifurone in both ascending auditory interneurons (AN1 and AN2) at their respective best frequencies (Fig. 6a). The calculated EC50 (effective concentration with 50% effect magnitude) for 20 µl injections is for both neurons at about 11 µmol/l flupyradifurone, with an effect range (EC20-EC80) of 3–40 µmol/l for AN1 and 5–30 µmol/l for AN2. We also compared the dose-dependent changes in spontaneous spiking of AN1 and AN2 individually (Fig. 6b). At the initial measurement and at relatively low flupyradifurone concentrations (10^− 7^ and 10^− 6^ mol/l), spontaneous spiking was low in AN1 and almost absent in AN2. With higher concentrations of flupyradifurone spontaneous spiking increased for both interneurons and peaked at 10^− 4^ mol/l, before it ceased completely at the highest tested concentration in most animals. Overall, the average spontaneous spiking activity was always higher for AN1 compared to AN2.

Finally, we calculated response-indices (auditory responsiveness vs. spontaneous activity; see methods for details) for the two auditory interneurons AN1 and AN2 at their respective best frequency after treatment with different flupyradifurone concentrations (Fig. 6c). Index values close to 1 correspond to relatively low spontaneous spiking activity and high spike responses to sound stimuli (spiking activity of AN1 and AN2 mainly codes sound signals) while values close to 0 indicate a similar spiking activity with and without sound stimulation (spike coding of sound signals is very low). In the ringer control group, the response-index was consistently high for both neurons (medians: 0.8 ± 0.03 for AN1 and 1.0 ± 0 for AN2) during the entire time of the experiment (Fig. 6c, left side). In the flupyradifurone treatment group, the response-index stayed high after 10^− 7^ and 10^− 6^ mol/l flupyradifurone injections (Fig. 6c, right side). For both neurons, there was a reduction of the response-index after injection with 10^− 5^ mol/l flupyradifurone (medians: 0.5 for AN1 and 0.7 for AN2), that further dropped after injection of 10^− 4^ mol/l flupyradifurone (medians: 0.2 for AN1 and 0.2 for AN2) when the spontaneous spiking increased while sound-induced responses decreased. For the highest flupyradifurone concentration tested (10^− 3^ mol/l), the response-index was in both cases at 0, as there was little to no spike generation in either auditory interneuron.

## Discussion

In this study we used surface suction electrodes at the cricket brain to investigate the effect of flupyradifurone on the spiking activity in the two ascending auditory interneurons AN1 and AN2. By comparison with intracellular recordings of AN1 and AN2 in the cricket brain, the action potentials responses of both individually identified interneurons could be detected as the large-amplitude spikes in suction electrode recordings covering the area of the auditory neuropile in the anterior protocerebrum (Schöneich et al. 2015; Kostarakos and Hedwig 2017). In contrast to intracellular AN1 and AN2 recordings, the suction electrode allowed us to monitor the spiking activity of these two individual interneurons over the relatively long time period required for the pharmacological experiments.

Our data show that flupyradifurone induces in both auditory interneurons, AN1 and AN2, a dose-dependent increase of their spontaneous spiking activity while their sound-evoked spike responses decreased. In our previous study we found that flupyradifurone intoxication caused, similar to classical neonicotinoids, a dose-dependent reduction of spike responses across the population of 15–20 ascending auditory interneurons in the locust neck connectives, while the responses of the auditory receptor neurons in the ears were not affected (Christian et al. 2025). These recordings in locusts led to the conclusion that the response failure of the ascending auditory interneurons by flupyradifurone might be mainly caused by an imbalance between inhibitory and excitatory synaptic inputs from intercalated local interneurons within the network. We also reported in locusts an increased spontaneous spiking activity in the connective recordings, but it was not possible to differentiate between individual auditory and non-auditory interneurons due to the experimental design (Christian et al. 2025). Contrary to the 3-layered auditory pathway in the locusts’ thoracic ganglia (Vogel et al. 2005; Hildebrandt et al. 2009), in field crickets the ascending auditory interneurons AN1 and AN2 receive monosynaptic excitatory inputs from the auditory receptor neurons (Hennig 1988; Hirtz and Wiese 1997; Imaizumi and Pollack 2005) and inhibition via the local ON1 neuron (Selverston et al. 1985; Horseman and Huber 1994). With the suction electrode at the cricket brain, here we selectively recorded the action potentials of AN1 and AN2 as extracellular spikes with a distinctively large amplitude. The data indicates that flupyradifurone caused a dose-dependent shift from pronounced sound-evoked spike bursts towards a predominantly spontaneous spiking activity in AN1 and AN2. At the highest tested dosage (20 µl of 10^− 3^ mol/l flupyradifurone) we recorded an almost complete reduction of the spontaneous spiking and sound-induced spike responses for both ascending auditory interneurons (Figs. 3, 5), similar to our previous recordings in locusts (Christian et al. 2025). Flupyradifurone had an immediate and long-lasting effect on auditory responses (starting a few minutes after injection and lasting for at least one hour; Fig. S1), which is in line with the relatively fast mode of action advertised for flupyradifurone in sucking pests (Nauen et al. 2015). Overall, AN1 and AN2 were equally affected by the flupyradifurone treatment, as we measured very similar dose-dependent effects on spontaneous spiking and sound-induced responses for both neurons (Figs. 5, 6).

In field crickets, the brain receives auditory input from two pairs of ascending auditory interneurons (AN1 and AN2), which code different behaviourally relevant sound information in their spiking pattern (Schöneich 2025). The sound frequency components of the acoustic signals are categorically encrypted on the basis of the labelled-line principle (Wyttenbach et al. 1996), which is hard-wired by the synaptic input connections of AN1 and AN2. For both interneurons, the amplitude of the sound signal is coded in the response by instantaneous spike rate (Schöneich 2025). With our data we calculated response-indices as simple indicator for how well sound information is coded in the recorded spiking activity with increasing insecticide concentration (Fig. 6c). The dose-dependent increase of spontaneous spiking activity was accompanied by a decrease of spike responses to sound stimuli in the two ascending auditory interneurons (Figs. 3, 5, 6). Both effects will lower the response-index, which makes it more difficult for the postsynaptic brain neurons to reliably detect and decode temporal stimulus features that are encoded in the spike trains. With increasing insecticide dosages, the response-index declined down to almost 0 for both interneurons at the two highest concentrations tested (Fig. 6c). After 20 µl injection of 10^− 4^ mol/l flupyradifurone the relatively high spontaneous activity equals the reduced auditory responses, whereas after 20 µl injection of 10^− 3^ mol/l both, spontaneous spiking and auditory responses, were equally low. Such a relatively low signal to noise ratio will be obstructive for precise coding of onset, timing and duration of sound pulses. AN1 is the relay neuron between low-frequency auditory receptors of the ear and the pulse pattern recognition network in the brain (Schöneich et al. 2015; Clemens et al. 2021). Therefore, an accurate representation of the calling song pattern in the spike responses of AN1 is crucial for mate finding behaviour (Schöneich 2020). AN2, on the other hand, is assumed to be a relay neuron between high-frequency auditory receptors and yet unidentified spike burst detection neurons of the brain (Marsat and Pollack 2010, 2012). The bilateral activity differences are used for sound localization, as intracellular electrical stimulation of AN2 on one side elicits directional avoidance steering (Nolen and Hoy 1984; Samson and Pollack 2002). An increase in the spontaneous spiking activity, as we recorded for AN1 and AN2 after flupyradifurone treatment, can mask the relevant signal features needed in the brain circuits for auditory pattern recognition and sound source localization (Schöneich 2025).

Sensory pathways are usually dynamic detectors of relevant changes in the environment of the animal and therefore the neural processing involves different forms of activity-dependent adaptation mechanisms for automatic gain control, dynamic background subtraction and threshold adjustment, so that the spike response of a neuron depends on the previous activity (Sobel and Tank 1994; Hildebrandt et al. 2009). Repeated sound stimulation can cause pre- and postsynaptic depression along the auditory pathway and also transient intrinsic inhibition of individual interneurons (Sobel and Tank 1994; Zucker and Regehr 2002; Cook et al. 2003). In our experiments we recorded a moderate amount of spontaneous spiking activity in AN1 and lower spontaneous spiking activity in AN2 (Figs. 1b, 6b) that was mostly supressed during the sound stimulation sequences (Fig. 1d). However, the enhanced spontaneous spiking activity after flupyradifurone treatment was present with and without sound stimulation (Figs. 2, 4). By acting as a competitive agonist at nAChRs in the insect central nervous system, flupyradifurone alters the spiking activity of interneurons similar to classical neonicotinoids (Christian et al. 2025). Flupyradifurone binding at the nAChRs causes overactivation of the interneurons by sustained depolarization (Buckingham et al. 1997). Similar to the activity-dependent adaptation mechanism that had been demonstrated in the omega neurons of the cricket (Sobel and Tank 1994), each action potential generated by the ascending auditory interneurons may also add to an accumulating transient inhibition due to intrinsic calcium-mediated hyperpolarization of the dendrite. Such mechanisms, based on the spiking-related activation of voltage-gated calcium channels (Sobel and Tank 1994; Jepson et al. 2006; Thany 2025), can function as an automatic gain control that shifts the working range of the sensory neuron depending on the stimulus amplitude and background noise level. This way the enhanced spontaneous spiking activity in AN1 and AN2 after insecticide treatment would dynamically shift the response threshold of the interneurons which could plausibly explain the dose-dependent reduction of the spike responses upon sound stimulation in our recordings. Moreover, the inhibitory omega neurons are most likely also affected by flupyradifurone in a similar way, which will disturb the balance between excitatory and inhibitory synaptic inputs of AN1 and AN2. In summary, the responses of individual interneurons in a small neuronal network like the cricket auditory pathway can be affected in a complex manner by simultaneous disturbance of synaptic inputs of the neuron and intrinsic mechanisms (Taha et al. 2024).

For comparability between studies, we calculated semi-logarithmic dose-response curves based on a 4-parameter logistic model that was fitted with the data of sound-induced responses for AN1 and AN2 at their respective best frequencies (Fig. 6a). This model estimates the 50% effective concentration (EC50) for both interneurons at about 11 µmol/l for the 20 µl flupyradifurone injections on average per cricket, which corresponds to about 45 ng/g body mass. For ascending auditory interneurons in locusts, the EC50 was 0.35 µmol/l for 100 µl flupyradifurone injections, which is a dosage of 7 ng/g body mass (Christian et al. 2025). Considering the tenfold increase of flupyradifurone dosage with each of the subsequent injection steps in our experiments, the two dosage values for crickets and locusts are still in the same order of magnitude. However, susceptibility to the same insecticides can largely differ even between closely related insect species, as it has been shown for different bees (Blacquière et al. 2012; Kline et al. 2025). Variations in the nAChR-subunit composition may explain such species-specific differences (Lu et al. 2022). The nAChRs are composed of 5 subunits, that dictate the pharmacological properties of the receptors (Ihara et al. 2017). While the core genes of the receptor subunits are highly conserved across insect taxa, variable expression patterns impact the binding affinity of the same insecticide between insect species (Jones and Sattelle 2010; Taillebois et al. 2018). Moreover, body size, metabolism, sex and many other factors can impact the efficacy of insecticides (Uhl et al. 2016; Kline et al. 2025). Considering the immense diversity of insect species in combination with the great variety of insecticides, the lack of standardised test methods and reporting norms still poses a major problem for the comparability between different studies (Nagloo et al. 2024).

## Supplementary Information

Below is the link to the electronic supplementary material.

Supplementary Material 1

## Data Availability

The data generated and analysed during the study are available from the corresponding authors on reasonable request.
