# Supplementary material for: Flupyradifurone impairs the processing of sound signals by the ascending interneurons AN1 and AN2 that convey auditory information to the cricket brain
Source: J Comp Physiol A Neuroethol Sens Neural Behav Physiol. 2026 May 11;212(4):617–32. doi: 10.1007/s00359-026-01814-4 (PMC13396037; doi:10.1007/s00359-026-01814-4)
Supplement: Supplementary file 1 — Supplementary Material 1 [file 359_2026_1814_MOESM1_ESM.pdf]

Flupyradifurone impairs the processing of sound signals by the ascending interneurons AN1 and AN2 that convey auditory information to the cricket brain

Marcelo Christian, Manuela Nowotny and Stefan Schöneich

Friedrich Schiller University Jena, Institute for Zoology and Evolutionary Research, Erbertstraße 1, 07743 Jena, Germany

Supplementary material:

Table S1: All p-values for sound-induced spike responses and spontaneous spiking activity of the two ascending auditory interneurons AN1 and AN2 combined for the different flupyradifurone treatments compared to the corresponding ringer controls (pairwise Wilcoxon comparison with Benjamini-Hochberg adjustment). Significant differences ($p<0.05$) correspond to the asterisks in Fig. 3 of the main text.

stimulus		p-values for 20 µl injections of different flupyradifurone concentrations					
frequency in kHz	amplitude in dB SPL	0 mol/l	10^{-7} mol/l	10^{-6} mol/l	10^{-5} mol/l	10^{-4} mol/l	10^{-3} mol/l
5	70	0.92	0.9	0.97	0.008	2.51×10^{-7}	7.59×10^{-6}
	75	0.92	0.9	0.97	0.004	9.49×10^{-6}	1.01×10^{-5}
	80	0.92	0.9	0.79	0.03	6×10^{-7}	1.19×10^{-5}
10	70	0.92	0.9	0.69	0.004	1.25×10^{-5}	7.59×10^{-6}
	75	0.92	0.9	0.69	3.93×10^{-4}	8.87×10^{-6}	7.59×10^{-6}
	80	0.92	0.9	0.69	8.55×10^{-4}	1.25×10^{-7}	7.59×10^{-6}
15	70	0.92	0.9	0.79	0.004	1.43×10^{-5}	1.38×10^{-5}
	75	0.92	0.9	0.69	0.004	1.67×10^{-7}	7.59×10^{-6}
	80	0.92	0.9	0.69	0.004	1.25×10^{-7}	7.59×10^{-6}
20	70	0.92	0.9	0.69	0.004	5.12×10^{-4}	1.33×10^{-5}
	75	0.92	0.9	0.69	0.004	1.37×10^{-5}	7.59×10^{-6}
	80	0.92	0.9	0.69	0.004	8.87×10^{-6}	7.59×10^{-6}
30	70	0.92	0.9	0.69	0.07	1.75×10^{-5}	1.19×10^{-5}
	75	0.92	0.9	0.97	0.03	7.5×10^{-5}	1.38×10^{-5}
	80	0.92	0.9	0.69	0.03	1.43×10^{-5}	3.94×10^{-5}
no stimulus		0.07	0.3	0.02	7.98×10^{-53}	9.96×10^{-53}	0.38

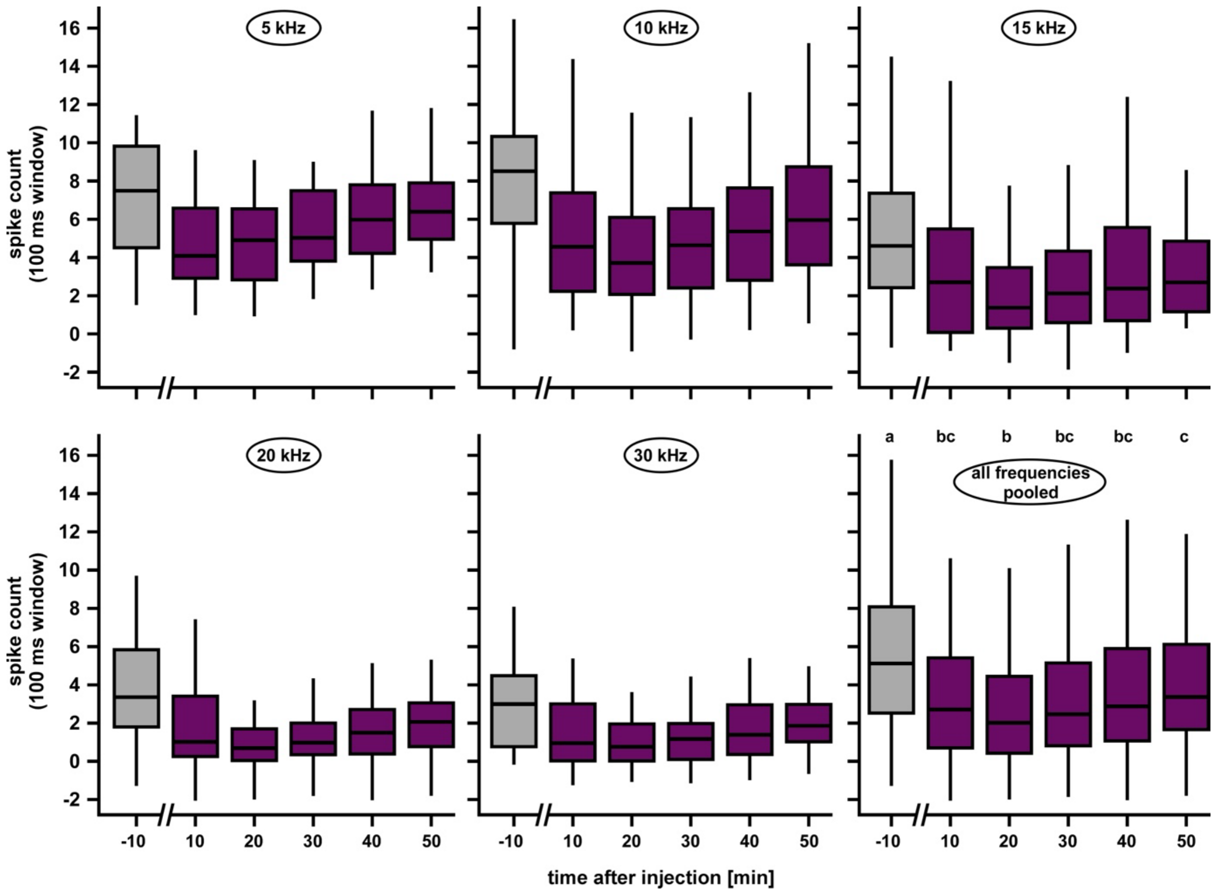

Figure S1: Time course of flupyradifurone effect on spike responses in the two ascending auditory interneurons AN1 and AN2 combined. Spike responses to 20 ms sound pulses at 3 different amplitudes (70, 75, 80 dB SPL) within a 100 ms time window after stimulus onset are shown before (grey) and five times in 10 min intervals after 20 µl injection of 10^{-5} mol/l flupyradifurone (purple). The graphs show the responses for each tested sound frequency (5, 10, 15, 20 and 30 kHz; n=30, N=10 each) and also the pooled response plot for all sound frequencies (lower right corner). Pooled data plot: Note the initial drop of spike responses within 10 min after the injection did not significantly change compared to the next four consecutive measurements in the pooled data of all frequencies (n=150, N=10). All spike responses were compared pairwise using the Wilcoxon test (‘rstatix’ R package version 0.7.1, p-values were Bonferroni adjusted). Different letters indicate significant differences ($p<0.05$) and same letters indicate no significant differences ($p>0.05$).
